# Prenatal Detection of Congenital Heart Diseases Using Echocardiography: 12-Year Results of an Improving Program With 9782 Cases

**DOI:** 10.3389/fpubh.2022.886262

**Published:** 2022-05-13

**Authors:** Yanqiu Ou, Michael S. Bloom, Jinzhuang Mai, Melissa Francois, Wei Pan, Xiaohua Xiao, Ximeng Wang, Zhiqiang Nie, Yanji Qu, Xiangmin Gao, Yong Wu, Xiaoqing Liu, Jian Zhuang, Jimei Chen

**Affiliations:** ^1^Department of Cardiac Surgery, Guangdong Cardiovascular Institute, Guangdong Provincial People's Hospital, Guangdong Academy of Medical Sciences, Guangzhou, China; ^2^Department of Global and Community Health, George Mason University, Fairfax, VA, United States; ^3^Department of Biomedical Sciences, University at Albany, State University of New York, Albany, NY, United States; ^4^Department of Pediatric Cardiology, Guangdong Cardiovascular Institute, Guangdong Provincial People's Hospital, Guangdong Academy of Medical Sciences, Guangzhou, China; ^5^Department of Cardiology, Boai Hospital of Zhongshan, Zhongshan, China

**Keywords:** congenital disease, birth defects, echocardiography, prenatal diagnosis, program, termination of pregnancy

## Abstract

**Background:**

A provincial program combining the effect of a government investment in prenatal screening and a specialized cardiac center was introduced in 2004, to improve prenatal diagnosis by echocardiography for congenital heart diseases (CHDs) in the Guangdong Registry of Congenital Heart Disease, China.

**Objectives:**

To evaluate the effects of this program on the prenatal diagnosis rate (PDR) by echocardiography and termination of pregnancy (TOP).

**Methods:**

A retrospective study from 2004-2015 included 9782 fetuses and infants diagnosed with CHDs. The PDR was calculated for major and minor CHDs during pre-, mid- and post-program time-intervals. Multivariable logistic regression was utilized to analyze the associations between program implementation and the timing of CHD diagnosis (prenatal vs. postnatal) by different hospital levels. The rate for TOP were also evaluated.

**Results:**

The PDR increased by 44% for major CHDs in the post-program interval relative to the pre-program interval. The three most frequently diagnosed subtypes prenatally were hypoplastic left heart syndrome (84%), double outlet right ventricle (83%) and severe pulmonary stenosis (82%). Participants with a high school education experienced a greater increase in PDR than those without a high school education. The odds for a prenatal vs. a postnatal diagnosis for major CHD were greater after introduction of the program than before (adjusted odd ratio= 20.95, 95% CI:2.47, 178.06 in secondary hospitals; and adjusted odd ratio=11.65, 95% CI:6.52, 20.81 in tertiary hospitals). The TOP rate decreased from 52.3% pre-program to 19.6% post-program among minor CHD fetuses with a prenatal diagnosis (*P* for trend =0.041). A lower proportion of TOP were attributed to minor CHDs after the program.

**Conclusions:**

The program combining the advantages of government investment and a specialized cardiac center appeared to increase the PDR by echocardiography for CHDs in an unselected population. The TOP rate among minor cases with prenatal diagnosis declined significantly after implementation of the program.

## Introduction

Congenital heart diseases (CHDs) remain the most common congenital malformations, with a global incidence of 6-12/1000 live births (1). CHDs account for 46% of all deaths due to congenital malformations and 3% of all infant deaths (2). ~20–30% of CHDs are “major”, defined as potentially life threatening and requiring surgery within the first year of life. Timing of diagnosis is a crucial factor for the prognosis of major CHD (3–5). Prenatal diagnosis of CHD can lead to timely delivery of medical treatment that may improve clinical outcomes and decrease neonatal morbidity and mortality (6). Over the past two decades, a great deal of emphasis has been placed on prenatal echocardiographic screening for CHDs, increased provider training, and establishing a national screening program (7).

Guangdong Cardiovascular Institute in Guangdong, China, initiated a program for improving the early diagnosis of CHDs with the support of the provincial government in 2004. Government policies promoted screening for congenital abnormalities, provincial networking, and professional training to improve detection, with the aim to improve CHD detection. Before 2006, sparse resources and few skilled ultrasonographers limited ultrasound screening for fetal abnormalities to evaluate obstetric complications or other high-risk pregnancies (8). However, many CHDs occur among “low risk” populations, mothers without known risk factors (9). Therefore, population-based CHD screening strategies have grown worldwide. Therefore, with the support of the provincial government, Guangdong Cardiovascular Institute established the Guangdong Registry of CHD (GRCHD) in 2004. The GRCHD provides professional education and training in early CHD diagnosis, and to offer medical priority for diagnosis to major cases, also to streamline CHD reporting across a network of 40 hospitals in Guangdong province.

In this report, we primarily aimed to assess the impact of the GRCHD program on the prenatal detection rate (PDR) and timing of diagnosis for CHDs, in an unselected population. We also examined the influence of the program on the termination of pregnancy (TOP) in the GRCHD.

## Materials and Methods

### Provincial Improving Program for Early Diagnosis of CHD

The provincial improving program included three major components and was summarized into “4P” Mode (Supplementary Figure 1):

#### Professional and Place

The specialized cardiac center, Guangdong Cardiovascular Institute, established and maintained a network of GRCHD across the province with the support of the Guangdong Department of Health, since 2004, aiming to unify the diagnosis and treatment of CHD. The network included the largest local hospitals and maternal and child care centers from various geographic areas across Guangdong province. Beginning with 19 surveillance sites at inception, the network gradually expanded to 39 sites in 20 cities at present, including primary, secondary, and tertiary hospitals (10, 11). The surveillance sites screen women and diagnose CHDs, which are reported to Guangdong Cardiovascular Institute in a standardized fashion. Guangdong Cardiovascular Institute, a specialized cardiac center in Guangzhou city, the provincial capital, is the coordinating and technical support center for the network. The coordinating center oversees the GRCHD, provides technical training, support, and quality control, and reports data to the Guangdong Department of Health. In addition, a CHD referral network ensures priority treatment for complicated and uncertain cases. Suspected CHDs are referred to Guangdong Cardiovascular Institute, where pediatric cardiologists validate the prenatal diagnosis; karyotyping is routinely recommended. After birth, newborns are evaluated by an obstetrician, pediatrician, or cardiologist before discharge, or within 72 h, to determine whether further investigation for potential CHD was required. All CHD cases are confirmed by B-mode echocardiography, cardiac catheterization, surgery, autopsy, or necropsy in the case of stillbirth. A 1-year followed up for the cases were conducted.

#### Promotion

Guangdong Cardiovascular Institute offers annual technical and standardization training on CHDs for staff from all network sites, including echocardiography knowledge and skills, fetal and infant cardiac anatomy, prenatal counseling, clinical management of CHDs, and intervention strategies. Completion of a 3-month intensive pediatric echocardiography in-service training program, offered by Guangdong Cardiovascular Institute, is required for the key echocardiographer from each network site. Guangdong Cardiovascular Institute also offers on-site and off-site technical consultation to network sites, provides timely feedback on referral cases, and helps improve diagnostic skills at lower-level facilities.

#### Policy

Government recommended the population to congenital abnormality screening. In 2006, the Guangdong Province Health Department formally recommended that all pregnant women receive routine ultrasound examination, including screening for fetal congenital anomalies, between 18 and 24 weeks of gestation (8). The recommendations for a basic cardiac examination included a 4-chamber view during prenatal ultrasound and, when technically feasible, views of the outflow tracts. Updated guidelines for prenatal ultrasound examinations by the Chinese Doctor Association, Council on Ultrasound (2012) also included views of three vessels when technically feasible (12). According to the updated recommendations, examination via fetal echocardiography is warranted for abnormal obstetrical ultrasound, the family history of CHD, maternal diabetes, or *use of in vitro* fertilization, in accordance with the practice guidelines for the performance of fetal echocardiography (13). Afterwards, some cities where the network sites located in across the province have initiated government-funded prenatal screening for congenital abnormalities beginning in 2008. Besides, Practice Guidelines for the Performance of Fetal Echocardiography in Guangdong Province was issued by the Guangdong Eugenics Association Council on Congenital Heart Diseases to guide the prenatal couseling on CHDs (14).

### Study Design and Participants

This is a multicenter population-based descriptive study of livebirths and stillbirths from the GRCHD diagnosed with CHDs with ultrasound, with estimated dates of delivery from 2004-2015. The study was approved by the Ethics Committee of Guangdong Provincial People's Hospital (Reference number: GDREC2011135H). Patient consent were obtained.

### Data Collection

All CHDs in newborns and stillbirths are reported to the national registry system for all birth defect. More detailed information obtained from hospital medical records is provided to the GRCHD. The GRCHD also obtains information from mothers through face-to-face interviews and a structured, standardized questionnaire on CHD risk factors as previously reported (10).

### Case Subclassification

All CHD cases in the GRCHD were coded by two clinical epidemiologists, with more than 20 years of experience in pediatric and adult cardiology, based on the International Classification of Diseases, 10th Revision (ICD-10) (Q20.000-Q28.000). CHD was classified into major CHDs and minor CHDs based on previous reports with slight modification (15). Major CHDs included: (1) dextro-transposition of the great arteries (TGA); (2) hypoplastic left heart syndrome (HLHS); (3) pulmonary atresia (PA), with or without ventricular septal defect (VSD); (4) tetralogy of Fallot (ToF); (5) total anomalous pulmonary venous return (TAPVR); (6) tricuspid atresia (TA); (7) truncus arteriosus; (8) aorta defects, such as aortic coarctation, atresia/hypoplasia/interruption of the aortic arch, and severe aortic valve stenosis; (9) double outlet right ventricle (DORV), (10) Ebstein anomaly; (11) severe pulmonic stenosis; (12) single ventricle complex; and (13) complete and partial atrioventricular septal defect (AVSD). Minor CHDs included: (1) isolated septal defects, such as isolated VSD, isolated atrial septal defect (ASD), and a combination of VSD and ASD; and (2) all other specific defects. Those with only prenatal diagnosis of ASD as an isolated heart disease, preterm infants with isolated patent ductus arteriosus, cases with isolated patent foramen ovale younger than 1 year, and cases without specific diagnosis or only with mild valve lesions were not considered to be CHDs.

### Data Analysis

The 12-year period was divided into three time-intervals, 1) 2004-2005, was defined as the “pre-program” interval, as there were no provincial CHD diagnosis data available before 2004; 2) 2006-2010, was defined as the “mid-program” interval; and 3) 2011-2015, was defined as the “post-program” interval. Characteristics of the participants, PDR, and timing of diagnosis were described for each CHD subtype by different intervals. The PDR was calculated as number of prenatal diagnosis cases divided by the total cases, for different CHD group (i.e., major CHDs or minor CHDs), CHD subtypes, and different program time intervals. *P* for trend was calculated by Cochran-Armitage trend test to estimate changing PDR trends during the different program time intervals. Multivariable logistic regression models were used to estimate the odds of a prenatal vs. postnatal diagnosis for major CHDs in the pre-program and mid-program/post-program intervals, stratified by different hospital levels. Factors adjusted in the multivariable regression was those highly related to the prenatal detection of CHDs, such as maternal age (>35 / ≤ 35 years), domestic migrant population (people moving from rural or undeveloped areas to Guangdong areas for work without permanent city residence; yes/no), maternal education (more than high school/completion of high school/less than high school), total previous live births (0/1/≥2), family history of CHD (yes/no), maternal diabetes (including pregestational and gestational diabetes, yes/no), multiple gestation (yes/no), infant sex (female/male), extra-cardiac defects or chromosomal abnormality (yes/no), and fetus with suspected cardiac abnormality on basic obstetric ultrasound (yes/no). The multiplicative scale of interaction was calculated by evaluating the product term for time-intervals or other factors and hospital levels. The statistical significance of the product term was determined by using the Wald statistic.

Missing data for maternal age (*n* = 486), domestic migration status (*n* = 112), maternal education (*n* = 46) or maternal previous live births (*n* = 43) were not imputed during analysis. Data were analyzed using SAS (v.9.3; SAS Institute, Inc., Raleigh, NC USA). Statistical tests were two sided with a significance level of *P* < 0.05.

## Results

### Characteristics of CHD Cases

A total of 9782 cases were diagnosed with CHD, among 1046456 fetuses and infants during the study period (Table 1). Compared to the pre-program interval, CHD cases in the mid-program and post-program intervals were more likely to be a maternal age ≥35 years and nulliparous, and less likely to be domestic migrant population and education less than high school (*P* < 0.05). During the study period, an increasing number of diagnoses were made at secondary compared to tertiary hospitals. The proportion of CHD cases diagnosed by tertiary hospitals significantly decreased (*P* for trend <0.001) from 70% pre-program to 46% post-program, while the proportion of CHD cases diagnosed by secondary hospitals significantly increased (*P* for trend <0.001), from 29% pre-program to 51% post-program.

**Table 1:** Characteristics of congenital heart disease cases, 2004–2015.

		**Total (*N* = 9782)**	**Pre-program (*N* = 673)**	**Mid-program** **(*N* = 3331)**	**Post-program (*N* = 5778)**
Maternal age (years)^*^					
	<35	8316 (85)	504 (75)	2888 (87)	4924 (85)
	≥35	980 (10)	57 (9)	294 (9)	629 (11)
**Maternal ethnicity**					
	Minorities	221 (2)	22 (3)	73 (2)	126 (2)
	Han	9561 (98)	651 (97)	3258 (98)	5652 (98)
**Domestic migrant population** ^†^				
	Yes	2552 (26)	223 (33)	1011 (30)	1318 (23)
	No	7118 (74)	450 (67)	2318 (70)	4350 (77)
**Maternal education** ^**‡**^					
	More than high school	1521 (16)	79 (12)	497 (15)	945 (17)
	Completion of high school	3174 (33)	202 (30)	939 (28)	2033 (36)
	Less than high school	5041 (52)	392 (58)	1895 (57)	2754 (48)
Maternal previous parity^**^					
	0	6495 (67)	466 (69)	2311 (69)	3718 (65)
	1	2638 (27)	174 (26)	865 (26)	1599 (28)
	2	606 (6.2)	33 (4.9)	154 (4.6)	419 (7.3)
**Infant sex**					
	Male	4783 (49)	356 (53)	1646 (49)	2781 (48)
	Female	4632 (47)	313 (47)	1679 (50)	2640 (46)
**Hospital level**					
	Tertiary hospital	4798 (49)	469 (70)	1702 (51)	2627 (46)
	Secondary hospital	4639 (47)	192 (29)	1488 (45)	2959 (51)
	Primary hospital	345 (4)	12 (2)	141 (4)	192 (3)

*Missing data*,

* *486 cases*;

† *112 cases*;

‡ *46 cases*;

** *43 cases*.

### Time Trends of PDR by Echocardiography

**Figure 1:**
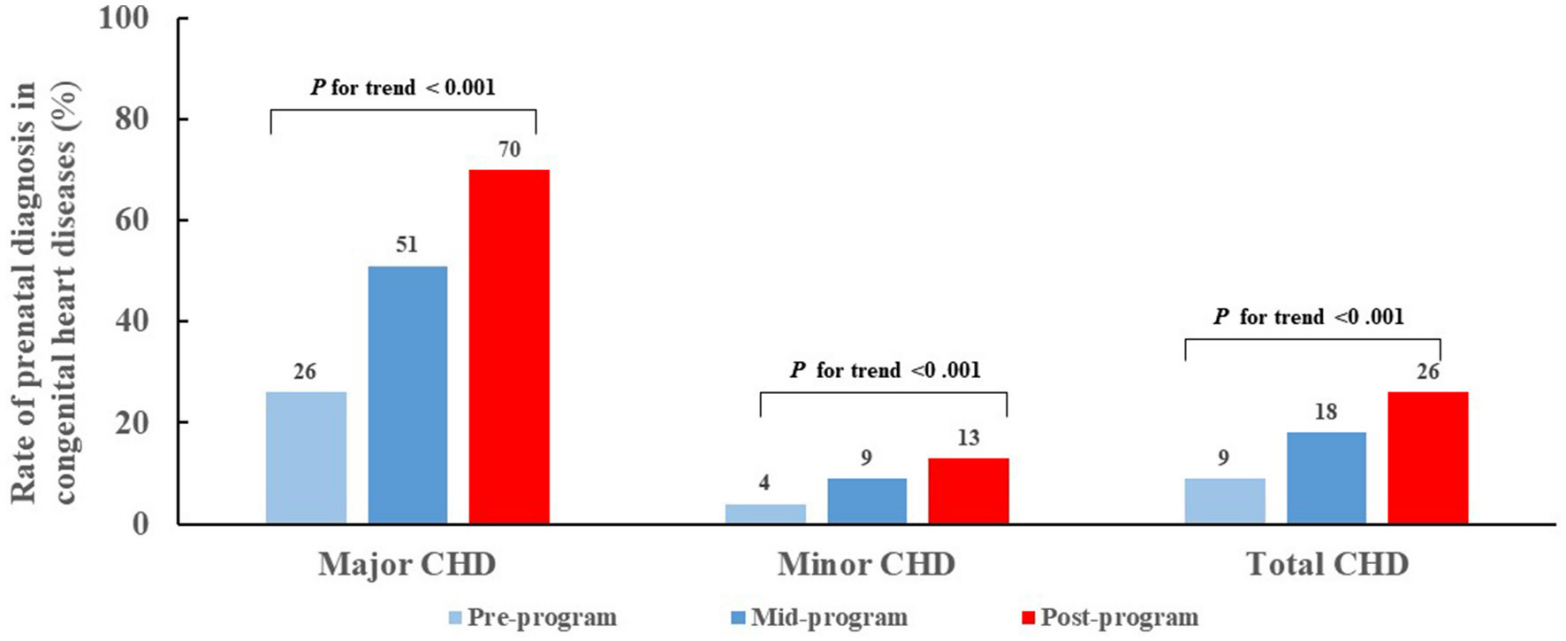
Trend for prenatal diagnosis rates for congenital heart diseases during pre-, mid- and post-program time intervals, by major and minor severity. *CHD*, congenital heart disease.

Of 9782 CHD cases diagnosed from 2004-2016, 2184 (22%) were diagnosed prenatally. Of note, the PDRs for total CHDs and CHD subgroups increased over time (Figure 1). Overall, CHD PDR increased 17 percentage points from 9% pre-program to 26% post-program (*P* for trend <0.001). The major CHD PDR increased 44 percentage points (from 26% pre-program to 70% post-program, *P* for trend <0.001) an average increase of 4 percentage points per year. The minor CHD PDR increased 9 percentage points from pre-program (4%) to post-program (13%) (*P* for trend <0.001).

### Prenatal Diagnosis by CHD Subtypes

Except for severe PS, all CHD subtypes experience dramatic increases in the PDRs (Table 2). The most significant PDR improvements were made for TA and aortic defects, with difference of more than 70% from the pre-program to the post-program intervals, followed by the ToF (60%), DORV (53%), PA (49%) and TGA (39%). Even with TAPVR, PDR increased by 32% throughout from the pre- program to the post-program intervals. The highest PDRs post-program were found for HLHS (84%), DORV (83%) and severe PS (82%). Almost half (49%) of TAPVR were detected prenatally. When stratified by the education level, we observed that the PDR improvement for major CHDs was most significant in participants completing education of high school, and then less than high school, and then higher than high school (Supplementary Figure 2).

**Table 2:** Prenatal congenital heart disease diagnoses, by subtype, 2004–2015.

		**Total**	**Pre-program**	**Mid-program**	**Post-program**	
		**n (PDR)**	**n (PDR)**	**n (PDR)**	**n (PDR)**	***P* for trend**
**Major CHD**	**2155 (61)**	**152 (26)**	**693 (51)**	**1310 (70)**	**<0.001**
	**TGA**	**485 (58)**	**40 (28)**	**167 (50)**	**278 (67)**	**<0.001**
	**HLHS**	**144 (80)**	**4 (50)**	**39 (72)**	**101 (84)**	**0.028**
	**PA**	**126 (56)**	**13 (15)**	**40 (55)**	**73 (64)**	**0.003**
	**TAPVR**	**86 (48)**	**6 (17)**	**29 (52)**	**51 (49)**	0.378
	**ToF**	**407 (50)**	**32 (9)**	**129 (26)**	**246 (69)**	**<0.001**
	TA	35 (60)	2 (0)	21 (57)	12 (75)	0.067
	Truncus arteriosus	108 (51)	12 (33)	33 (49)	63 (56)	0.158
	**Aortic defects** ^*^	**174 (71)**	**3 (0)**	**45 (60)**	**126 (76)**	**0.002**
	**DORV**	**212 (81)**	**10 (30)**	**70 (84)**	**132 (83)**	**0.023**
	Ebstein anomaly	53 (76)	4 (75)	21 (71)	28 (79)	0.654
	Severe PS	38 (84)	2 (100)	14 (86)	22 (82)	0.529
	Single ventricle	62 (60)	6 (50)	18 (50)	38 (66)	0.259
	**AVSD** ^†^	**225 (54)**	**18 (33)**	**67 (36)**	**140 (66)**	**<0.001**
**Minor CHD**	**7627 (11)**	**521 (4)**	**2638 (9)**	**4468 (13)**	**<0.001**
	**Isolated septal defects** ^**‡**^	**3279 (12)**	**240 (5)**	**1034 (11)**	**2005 (14)**	**<0.001**
	**Other specified defects**	**4348 (10)**	**281 (3)**	**1604 (8)**	**2463 (13)**	**<0.001**
**Total CHD**	**9782 (22)**	**673 (9)**	**3331 (18)**	**5778 (26)**	**<0.001**	

*ASD, atrial septal defect; AVSD, atioventricular septal defect; CHD, congenital heart disease; DORV, double outlet right ventricle; HLHS, hypoplastic left heart syndrome; PA, pulmonary atresia; PDR, prenatal detection rate; PS, pulmonary stenosis; TA, tricuspid atresia; TAPVR, total anomalous pulmonary venous return; TGA D-, Transposition of the great arteries; ToF, tetralogy of fallot; VSD, ventricular septal defect*.

* *Aorta defects includes aortic coartation, atresia/hypoplasia/interruption of the aortic arch and severe aortic valve stenosis*.

† *AVSD includes complete and partial subtypes*.

‡ *Isolated septal defects include isolated VSD, isolated, and combination of VSD and ASD. The bold values indicates the trend test for PDR in different time intervals is with statistical significance*.

**Figure 2:**
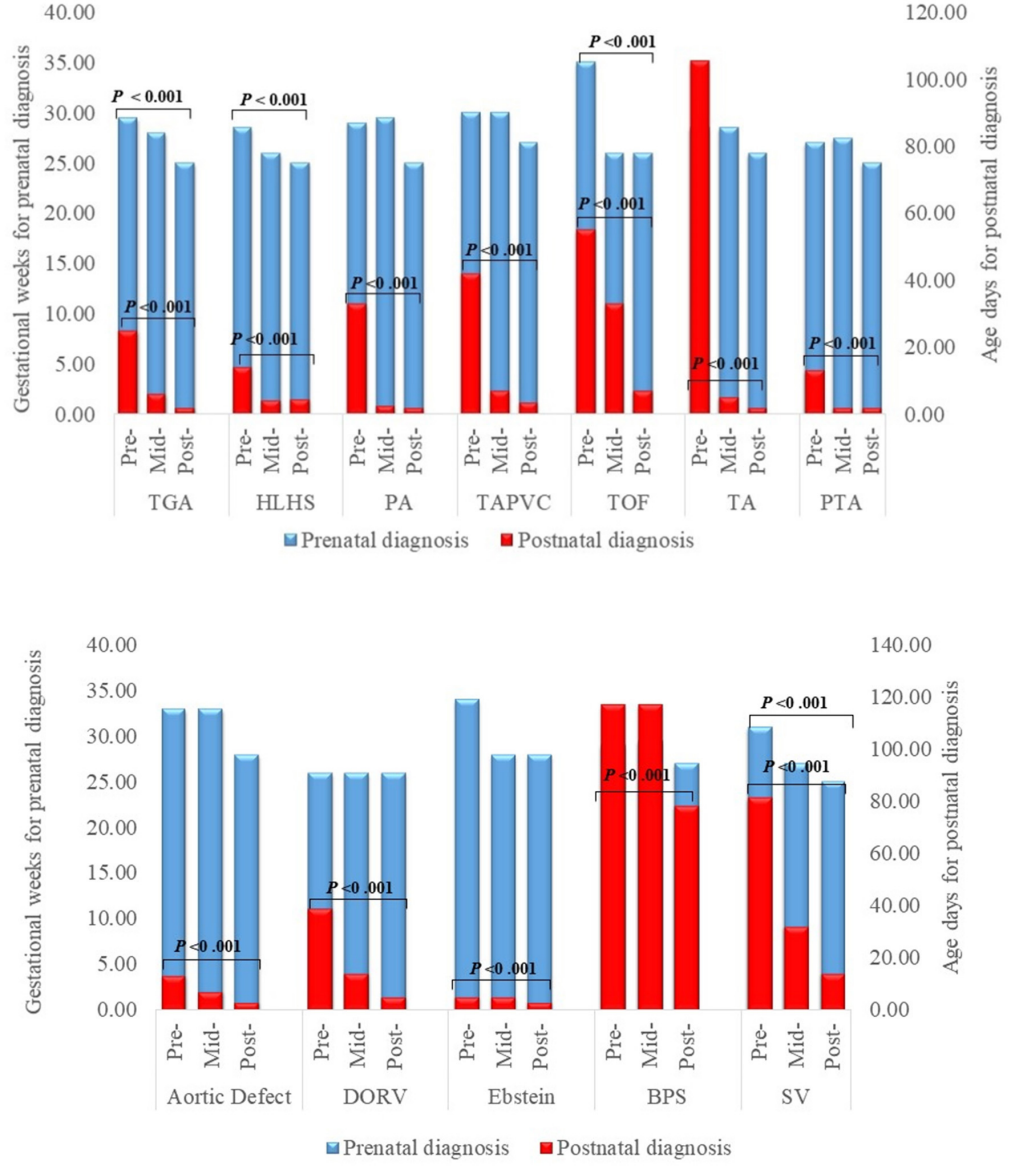
Timing of prenatal and postnatal diagnosis for major congenital heart defects. *DORV* Double Outlet Right Ventricle, *HLHS* Hypoplastic Left Heart Syndrome, *PA* Pulmonary Atresia, *PDR* Prenatal Detection Rate, *PS* Pulmonary Stenosis, *TA* Tricuspid Atresia, *TAPVR* Total Anomalous Pulmonary Venous Return, *TGA* D-Transposition of the Great Arteries, *ToF* Tetralogy of Fallot.

The times of prenatal and postnatal diagnosis also greatly advanced as shown in Figure 2. The median gestational week at prenatal diagnosis was 30 weeks pre-program and 25 weeks post-program for each specific subtype, with statistically significant differences for TGA, HLHS, ToF and SV (*P* for trend <0.001). Postnatal CHD diagnosis also advanced significantly (all *P* for trend <0.001), with most major post-program CHDs detected within three (TGA, PA, TAPVR, TA, PTA, aortic defect, and Ebstein anomaly) to seven days (HLHS, ToF and DORV) after delivery.

### Odds of Prenatal Diagnosis for CHD in Different Time-Intervals and Population

**Figure 3:**
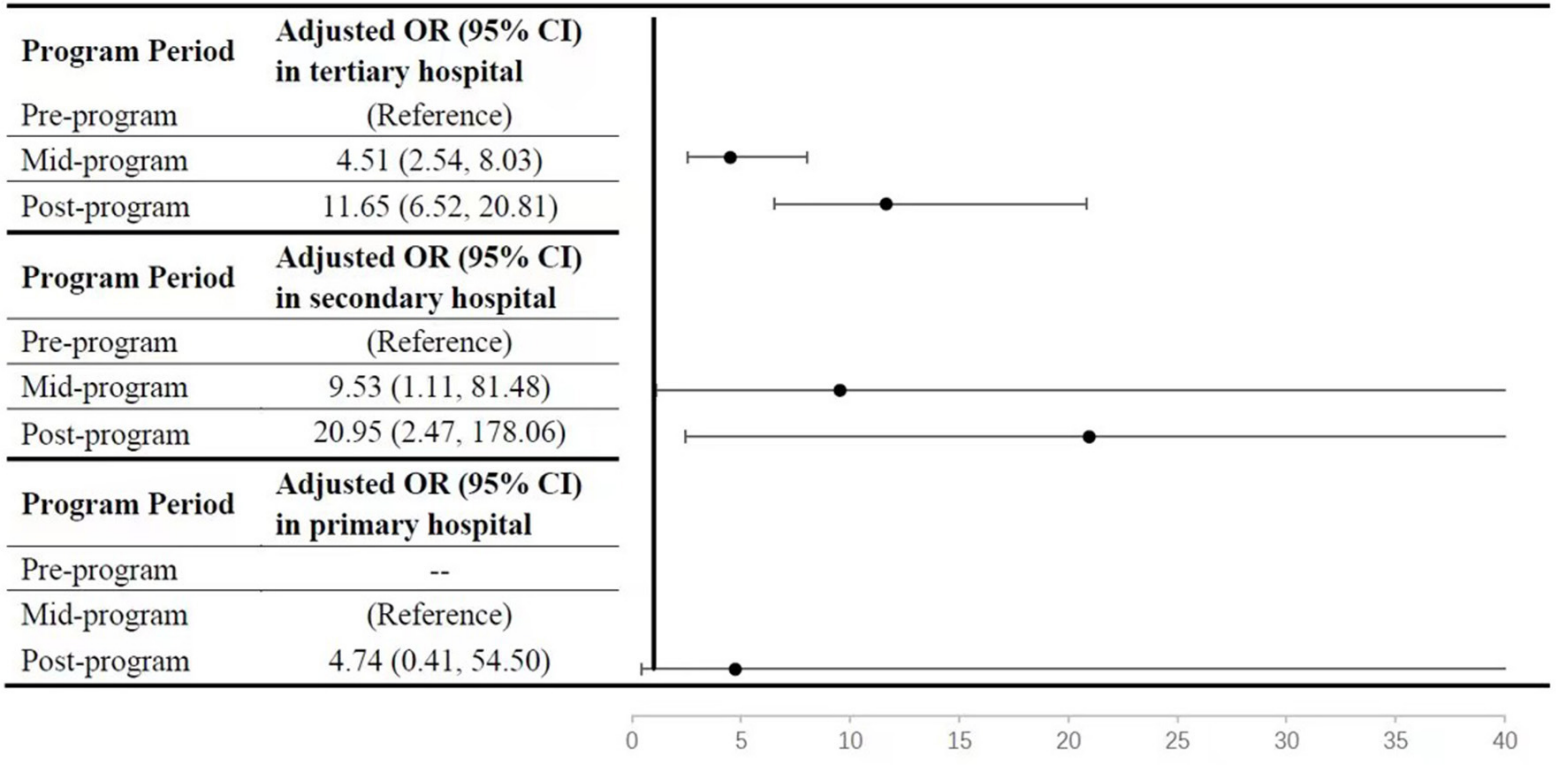
Multivariate logistic regression analysis for different time-interval associated with the prenatal diagnosis for major congenital heart diseases stratified by different hospital levels, 2004–2015. The multivariable models in different hospital stratification was adjusted by maternal age (>35 yrs and ≤ 35 yrs), domestic migrant population (yes/no), maternal education (more than high school/completion of high school/less than high school), total previous live births (0/1/≥2), family history of CHD (yes/no), maternal diabetes (yes/no), maternal hypertension (yes/no), infant sex (female/male), extra-cardiac/aneuploidy/genetic syndrome (yes/no), and fetus with suspected cardiac abnormality on basic obstetric ultrasound (yes/no). *OR*, Odds Ratio; CI, confidence interval.

To further elucidate the contribution of the provincial improving program to the advancing major CHD PDR, multivariable logistic regression was used to analyzed the odds of receiving a prenatal vs. postnatal diagnosis for major CHD in the different program intervals stratified by different hospitals. As shown in Figure 3, compared to cases in the pre-program period, major CHD cases in mid-program and post-program periods had higher odds of prenatal diagnosis. The increment of adjusted OR (aOR) by time-interval was strongest in the secondary hospitals (aOR=9.53, 95% CI: 1.11, 81.48 in mid-program period; and aOR=20.95, 95% CI:2.47, 178.06 in post-program period, respectively), and then the tertiary hospitals (aOR=4.51, 95% CI: 2.54, 8.03 in mid-program period; and aOR=11.65, 95% CI:6.52, 20.81 in post-program period, respectively). The interaction between time-intervals and hospital levels was statistically significant (P < 0.001). In addition, different level centers had particular factors associated with increased PDR (Supplementary Table 1). Factors interacted with hospital level included domestic migrant population, primiparous, female infant, and fetus with extra-cardiac/aneuploidy/genetic syndrome (P for multiplicative interaction <0.01). According to the performance guideline in the GRCHD (13), indicators for fetal heart screening include the presence of abnormal obstetrical ultrasound results, the presence of a family history of CHD, maternal diabetes, or *use of in vitro* fertilization, and so on. Except for the major CHDs, total CHDs and minor CHDs with these indicators were more easily to be detected prenatally (*P* < 0.001, Table 3). However, only about 8% of the cases had these factors, like the presence of abnormal obstetrical ultrasound results, the presence of a family history of CHD, maternal diabetes, or *use of in vitro* fertilization, and so on.

**Table 3:** Prenatal CHD diagnosis rates by presence of additional CHD risk factors.

	Risk Factor^*^Not Present, *N* (%)	**PDR**	Risk Factor^*^Present, *N* (%)	**PDR**	***P*-value for PDR**
Total CHD	1991, 92%	22%	177, 8%	29%	<0.001
Major CHD	1214,92%	61%	100, 8%	60%	0.763
Minor CHD	777, 91%	11%	77, 9%	18%	<0.001

* *Risk factors includes the presence of abnormal obstetrical ultrasound results, the presence of a family history of CHD, maternal diabetes, or use of in vitro fertilization, and so on*.

### Termination of Pregnancy

**Figure 4:**
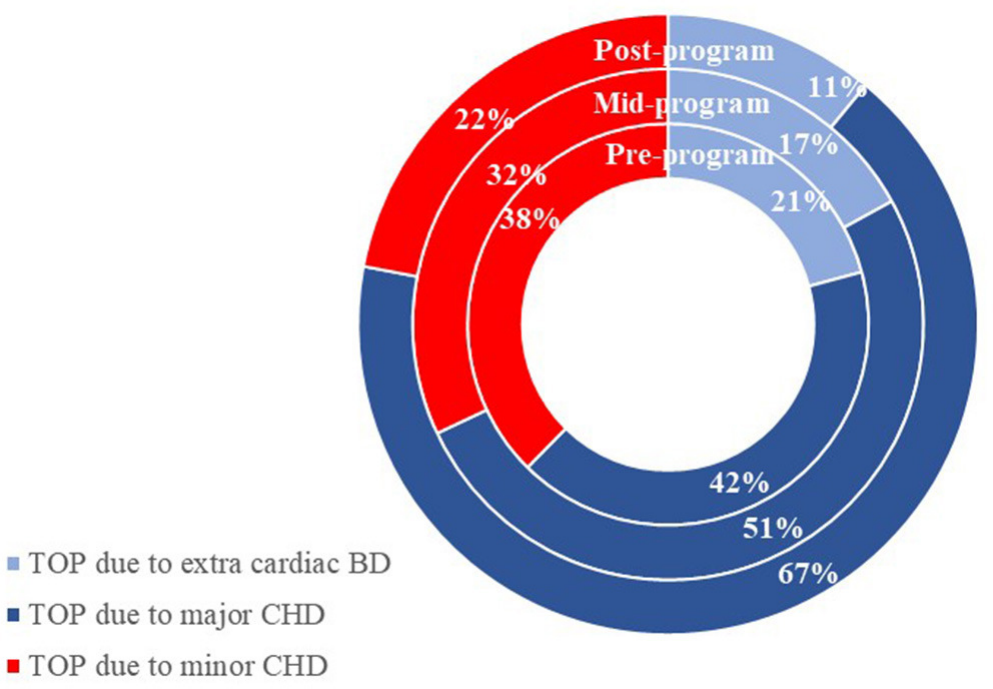
The distribution of the indicators for termination of a pregnancy with a prenatal congenital heart disease diagnosis. *BD*, birth defect; *CHD*, congenital heart disease; *PDR*, prenatal detection rate; *TOP*, termination of pregnancy.

During the study period, the rate for TOP deceased from 40% pre-program to 30% post-program among total CHD fetuses with prenatal diagnosis (*P* for trend = 0.060), while the TOP rate dropped from 52.3% to 19.6% among minor CHD fetuses with prenatal diagnosis (*P* for trend = 0.041). The TOP attributed largely to major CHDs, progressively lower proportion of TOP were due to minor CHDs after the program (from 38% pre-program to 22% post-program, *P* for trend = 0.028, Figure 4).

## Discussion

### Main Findings

In this large population-based study, we found that implementation of a provincial improving program for the early diagnosis of CHDs was associated with greater CHD PDRs, and fewer pregnancy terminations resulted from minor CHD. Following program implementation, PDRs increased almost 50% for major CHDs, with 70% of major defects detected prenatally. Participants with a high school education experienced a greatest increase in PDR than those without a high school education. While major CHD subtypes continued to be terminated, termination of minor CHD subtypes diminished over time. To the best of our knowledge, this is the first large population-based study to investigate the effect of a regional program on CHD in China.

### Interpretation

#### PDR Over Time and Subtype Detection

The largest increase in PDRs with the progress of the program, 2 years after the start of the improving program, which verified the effect of the program after a learning period for the ultrosonographers. Our findings were was consistent with previous studies of Netherlands, which reported the PDRs for severe CHDs (*n* = 1912) increased from 35.8% in 2002 to 59.7% in 2012 after the 2007 introduction of a national prenatal screening program (7). While the pre- and mid-program PDRs of major CHDs were lower in the current study than reported for developed countries, post-program PDRs were similar or exceeded rates from developed countries. For example, the post-program prenatal HLHS PDR in the current study (84%) was similar to the 87.9% reported in Massachusetts, and higher than the 50% reported for the multisite U.S. National Birth Defects Prevention Study (4, 16). In our study, the detection rate for TAPVR was higher than some studies from developed nations reporting no prenatally detected cases (15).

We also found a noteworthy advancement of gestational age diagnosis for prenatal CHDs after program implementation. The ~5 weeks of earlier prenatal diagnosis for major CHDs allows more time for the family to plan the delivery at a higher or specialized medical center, or to receive intervention *in utero* to prevent deterioration. For postnatal major CHDs diagnosed after program implementation, we also found a median diagnosis time 3 to 7 days earlier than pre-program which may allow for earlier and perhaps more effective treatment of very critical and severe lesions.

#### Role of The Provincial Improving Program on PDR Increase

Technical training and education is vital to improve the PDR. Fetal echocardiography, in experienced hands, has been reported to detect up to 90% of CHDs in low-risk population (9). However, given the spectrum and complexity of CHD pathologies, obstetrical ultrasound screening has a very low diagnostic yield (10%−26%) (17–19). Therefore, the standardized on-site and off-site training, that pediatric cardiologists provide to network hospital physicians and ultrasonographers, is important to improve the overall level of CHD diagnosis using ultrasound. The effectiveness of this technical training provided to network hospitals is supported by our results, which suggests a greater frequency of CHD cases reported by secondary hospitals in in the post-program interval, even exceeding the PDR of more specialized tertiary hospitals. And the multivariable logistic regression stratified by the hospital level further convinced that the improving program benefit the secondary and tertiary hospitals, and had significant public health implication.

Another important contributor was the inclusion of outflow tracts view and 3-vessels views, in addition to 4-chamber view, during prenatal ultrasound diagnosis. Theoretically, the 4-chamber view detects >50% of serious cardiac malformations when performed in mid-gestation. The addition of the outflow tracts and 3-vessel with trachea view increases sensitivity to as much as 90% (20–22). This can give explanation to our finding that, both PDRs with lesions visible and invisible on the 4-chamber view has significantly increased (Supplementary Figure 3). The gap between the two groups shortened, which implicated the detection of CHDs prenatally does not rely on the 4-chamber view.

Governmental recommendations are required to help allocate medical investment in congenital abnormalities screening, and standardized the clinicians daily work process. In addition, some cities across the region have initiated government-funded prenatal screening for congenital abnormalities beginning in 2008, which has likely boosted the number of low-risk pregnant mothers who have access to screening for cardiac defects. These not only benefited participants with high level education, and to a greater degree, also benefited those with high school education or less than high school.

#### Termination of Pregnancy

The TOP rate in our study population was much lower than that in Norther China (40% vs. 85%) (23). And notably, we were able to observe the TOP rate for minor case reducing to 19.6%, as well as fewer TOP attributing to minor CHDs post-program. We attribute these finding to the promotion of the improving program, which provided training not only on diagnosis skills, but also on standardized prenatal consultation. According to the Practice Guidelines for the Performance of Fetal Echocardiography in Guangdong Province (14), we strongly encouraged the family to give birth to the fetus with minor CHDs and defects that will have promising prognosis postnatally.

We also found that the overall and minor TOP rate for CHDs with prenatal detection in the current study was lower than our previously report about the specialized prenatal consultation service in our specialized center (40% vs. 58.1% for overall, 19.6% vs. 30.1% for minor CHDs) (24). The discrepancies were mainly due to different study population and design. The current study was population-based and demonstrate the “real world” data, validating the influence of the specialized prenatal consultation service in the generalized population.

### Strengths and Limitations

This study is one of the few and the largest studies to report a provincial program to improve the PDR across the region. The long study period enabled assessment of the program by different time intervals, and the large number of cases size enabled evaluation of PDR by different CHD subtypes. Also, we evaluated the change for TOP rate by program progress. In addition, each CHD case was individually reviewed by two clinical epidemiologists trained in pediatric and adult cardiology. Similar to several previous studies (25, 26), our definition of major CHD required documentation of clinical severity in addition to ICD code.

However, there were several potential limitations of the study. First, the GRCHD is not a province-wide registry, only covering about 12% newborns from the whole province. Therefore, it could not capture all CHD cases from the province. However, the GRCHD included the largest local hospitals and maternal and child care centers from each city across Guangdong province, which have the capability of detecting CHD prenatally or in newborns. We think the GRCHD could represent the overall level for diagnosing CHD across the whole province. Moreover, there was no control group without any program implementation, or a parallel data set for comparison. An ongoing project using two parallel population-based cohorts with and without program implementation, is conducted in Zhongshan City, Guangdong Province, which is expected to shorten this limitation. Several other factors may contribute to improved PDR, including affordability, social insurance schemes, and access to healthcare in general. It was a limitation that we did collect these data to evaluate their influence. Furthermore, the influence of differences in diagnosis time on later mortality, morbidity, and quality of life of the affected babies is not currently available. The long-term follow-up for these CHD patients and prognosis evaluation is required, to validate the effect of prenatal diagnosis.

## Conclusions

The results of this large multicenter study indicate that a program combining the advantage of government investment, and a specialized cardiac center is an effective means to improve the PDR of CHDs in an unselected population. The TOP rate among minor cases with prenatal diagnosis declined significantly with the program. We presume that similar programs will be effective for improving prenatal CHD diagnoses in other large Chinese provinces and in Asian countries with different levels of hospitals. Future studies will be necessary to evaluate the effectiveness of this approach in different populations.

## Data Availability Statement

The datasets presented in this article are not readily available, because the data was generated from different hospitals of the GDCHD network. We were not authorized to share the data outside the network. Requests to access the datasets should be directed to ouyanqiu@gdph.org.cn.

## Ethics Statement

The studies involving human participants were reviewed and approved by the Ethics Committee of Guangdong Provincial People's Hospital (Reference number: GDREC2011135H). Written informed consent to participate in this study was provided by the participants' legal guardian/next of kin.

## Author Contributions

YO and JC conceived and designed the study and designed the protocol. YO, JM, and MF conducted the statistical analysis. MB critically reviewed the manuscript. All authors contributed to the article and approved the submitted version.

## Funding

This work was supported by the National Key Research and Development Program [No. 2018YFC1002600], the National Natural Science Foundation of China [No. 81903287], the Natural Science Foundation of Guangdong Province [Nos. 2021A1515011445 and 2020A1515010743], Guangdong Provincial Key Laboratory of South China Structural Heart Disease [No. 2012A061400008] and Guangdong Provincial Clinical Research Center for Cardiovascular Disease [No. 2020B1111170011].

## Conflict of Interest

The authors declare that the research was conducted in the absence of any commercial or financial relationships that could be construed as a potential conflict of interest.

## Publisher's Note

All claims expressed in this article are solely those of the authors and do not necessarily represent those of their affiliated organizations, or those of the publisher, the editors and the reviewers. Any product that may be evaluated in this article, or claim that may be made by its manufacturer, is not guaranteed or endorsed by the publisher.

## Supplementary Material

Supplementary Figure 1 The diagrammatic drawing for the “4P” Mode to improve the diagnosis capability across the region.

Supplementary Figure 2 Prenatal diagnosis rate for congenital heart disease by maternal education, 2004-2015.

Supplementary Figure 3 Trend for prenatal diagnosis rates for congenital heart disease during pre-, mid- and post-program time intervals, by invisible or visible on the 4-chamber view of fetal ultrasound screening. ^*^*P* < 0.05 compared to the pre-program PDR in lesion invisible on 4-chamber view.

## Abbreviations

AVSD: atrioventricular septal defect
ASD: atrial septal defect
CHDs: congenital heart diseases
DORV: double outlet right ventricle
GRCHD: Guangdong Registry of CHD
HLHS: hypoplastic left heart syndrome
ICD-10: International Classification of Diseases, 10th Revision
PA: pulmonary atresia
PDR: prenatal detection rate
TA: tricuspid atresia
TAPVR: total anomalous pulmonary venous return
TGA: dextro-transposition of the great arteries
ToF: tetralogy of Fallot
TOP: termination of pregnancy
VSD: ventricular septal defect.

## References

[1] Donofrio MT Moon-Grady AJ Hornberger LK Copel JA Sklansky MS Abuhamad A. Diagnosis and treatment of fetal cardiac disease: a scientific statement from the American heart association. Circulation. (2014) 129:2183–242. 10.1161/01.cir.0000437597.44550.5d 24763516

[2] Zeng ZD Zhang HW Liu FL Zhang NN. Current diagnosis and treatments for critical congenital heart defects. Exp Ther Med. (2016) 11:1550–4. 10.3892/etm.2016.3167 27168772 PMC4840484

[3] Chang RK Gurvitz M Rodriguez S. Missed diagnosis of critical congenital heart disease. Arch Pediatr Adolesc Med. (2018) 162:969–74. 10.1001/archpedi.162.10.969 18838650

[4] Liberman RF Getz KD Lin AE Higgins CA Sekhavat S Markenson GR. Delayed diagnosis of critical congenital heart defects: trends and associated factors. Pediatrics. (2014) 134:e373–81. 10.1542/peds.2013-3949 25070301 PMC9923616

[5] Peterson C Dawson A Grosse SD Riehle-Colarusso T Olney RS Tanner JP. Hospitalizations, costs, and mortality among infants with critical congenital heart disease: How important is timely detection? Birth Defects Res A Clin Mol Teratol. (2013) 97:664–72. 10.1002/bdra.23165 24000201 PMC4473256

[6] Nelle M Raio L Pavlovic M Carrel T Surbek D Meyer-Wittkopf M. Prenatal diagnosis and treatment planning of congenital heart defects-possibilities and limits. World J Pediatr. (2009) 5:18–22. 10.1007/s12519-009-0003-8 19172327

[7] van Velzen CL Clur SA Rijlaarsdam ME Bax CJ Pajkrt E Heymans MW. Prenatal detection of congenital heart disease–results of a national screening program. Bjog-Int J Obstet Gy. (2016) 123:400–7. 10.1111/1471-0528.13274 25625301

[8] Health Department of Guangdong Province in China. Guideline for the performance of obstetric ultrasound examinations. Bulletin People's Gover Guangdong Provinc. (2006) 17:16–31.

[9] Stumpflen I Stumpflen A Wimmer M Bernaschek G. Effect of detailed fetal echocardiography as part of routine prenatal ultrasonographic screening on detection of congenital heart disease. Lancet. (1996) 348:854–7. 10.1016/S0140-6736(96)04069-X 8826811

[10] Ou YQ Mai JZ Zhuang J Liu XQ Wu Y Gao XM. Risk factors of different congenital heart defects in Guangdong, China. Pediatr Res. (2016) 79:549–58. 10.1038/pr.2015.264 26679154

[11] Qu YJ Liu XQ Zhuang J Chen GC Mai JZ Guo XL. Incidence of congenital heart disease: the 9-year experience of the Guangdong registry of congenital heart disease, China. PLoS ONE. (2016) 11:e0159257. 10.1371/journal.pone.0159257 27409588 PMC4943720

[12] Chinese Doctor Association Council on Ultrasound. Guidelines of prenatal ultrasound examinations. Chin J Med Ultrasound. (2012) 9:574–80.

[13] Guangdong Provincial People's, Hospital Guangdong Cardiovascular Institute, Guangdong Eugenics Association Council on Congenital Heart Diseases. Practice guidelines for the performance of fetal echocardiography in Guangdong province. Int Med Health Guid News. (2015) 21:739–40.

[14] Guangdong Provincial People's, Hospital Guangdong Cardiovascular Institute, Guangdong Eugenics Association Council on Congenital Heart Diseases. Practice Guidelines for the Performance of Prenatal Counseling on Fetal Congential Heart Diseases in Guangdong Province. Int Med Health Guid News. (2015) 21:1033–36.

[15] Olney RS Ailes EC Sontag MK. Detection of critical congenital heart defects: review of contributions from prenatal and newborn screening. Semin Perinatol. (2015) 39:230–7. 10.1053/j.semperi.2015.03.007 25979782 PMC4460982

[16] Ailes EC Gilboa SM Riehle-Colarusso T Johnson CY Hobbs CA Correa A. Prenatal diagnosis of nonsyndromic congenital heart defects. Prenatal Diag. (2014) 34:214–22. 10.1002/pd.4282 24222433 PMC4482476

[17] Ewigman BG Crane JP Frigoletto FD LeFevre ML Bain RP McNellis D. Effect of prenatal ultrasound screening on perinatal outcome: RADIUS study group. N Engl J Med. (1993) 329:821–7. 10.1056/NEJM199309163291201 8355740

[18] Garne E1 Stoll C Clementi M. Evaluation of prenatal diagnosis of congenital heart diseases by ultrasound: experience from 20 European registries. Ultrasound Obstet Gynecol. (2001) 17:386–91. 10.1046/j.1469-0705.2001.00385.x 11380961

[19] Tegnander E Eik-Nes SH Johansen OJ Linker DT. Prenatal detection of heart defects at the routine fetal examination at 18 weeks in a nonselected population. Ultrasound Obstet Gynecol. (1995) 5:372–80. 10.1046/j.1469-0705.1995.05060372.x 7552797

[20] Del Bianco A Russo S Lacerenza N Rinaldi M Rinaldi G Nappi L. Four chamber view plus three-vessel and trachea view for a complete evaluation of the fetal heart during the second trimester. J Perinat Med. (2006) 34:309–12. 10.1515/JPM.2006.059 16856821

[21] Kirk JS Riggs TW Comstock CH Lee W Yang SS Weinhouse E. Prenatal screening for cardiac anomalies: the value of routine addition of the aortic root to the four-chamber view. Obstet Gynecol. (1994) 84:427–31. 8058243

[22] Marek J Tomek V Skovránek J Povysilová V Samánek M. Prenatal ultrasound screening of congenital heart disease in an unselected national population: a 21-year experience. Heart. (2011) 97:124–30. 10.1136/hrt.2010.206623 21163892

[23] Yang XY Li XF Lv XD Liu YL. Incidence of congenital heart disease in Beijing, China. Chin med J (Engl). (2009) 122:1128–32. 19493457

[24] Qu YJ Chen JJ Han FZ Lin S Bell EM Pan W. Can we improve the perinatal outcomes and early postnatal survival of fetuses with congenital heart disease by initiating specialized prenatal consultation service? Clinics Mother Child Health. (2020) 17:360.

[25] Quartermain MD Pasquali SK Hill KD Goldberg DJ Huhta JC Jacobs JP. Variation in prenatal diagnosis of congenital heart disease in infants. Pediatrics. (2015) 136:e378–85. 10.1542/peds.2014-3783 26216324 PMC4844533

[26] Peterson C Ailes E Riehle-Colarusso T Oster ME Olney RS Cassell CH. Late detection of critical congenital heart disease among US infants: estimation of the potential impact of proposed universal screening using pulse oximetry. JAMA Pediatr. (2014) 168:361–70. 10.1001/jamapediatrics.2013.4779 24493342 PMC4470377

