# Supplementary figures and images for: Prenatal Detection of Congenital Heart Diseases Using Echocardiography: 12-Year Results of an Improving Program With 9782 Cases
Source: Front Public Health. 2022 May 13;10:886262. doi: 10.3389/fpubh.2022.886262 (PMC9136016; doi:10.3389/fpubh.2022.886262)

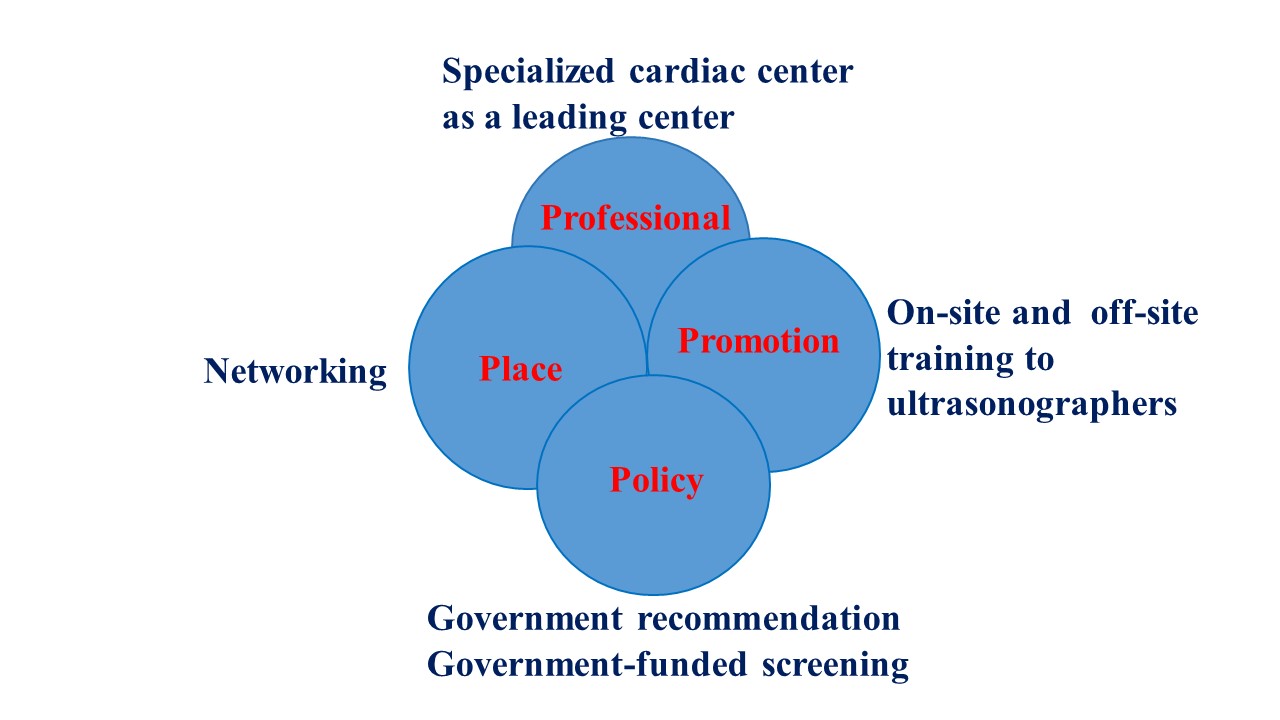

Supplement: Supplementary Figure 1 — The diagrammatic drawing for the “4P” Mode to improve the diagnosis capability across the region. [file Image_1.JPEG]

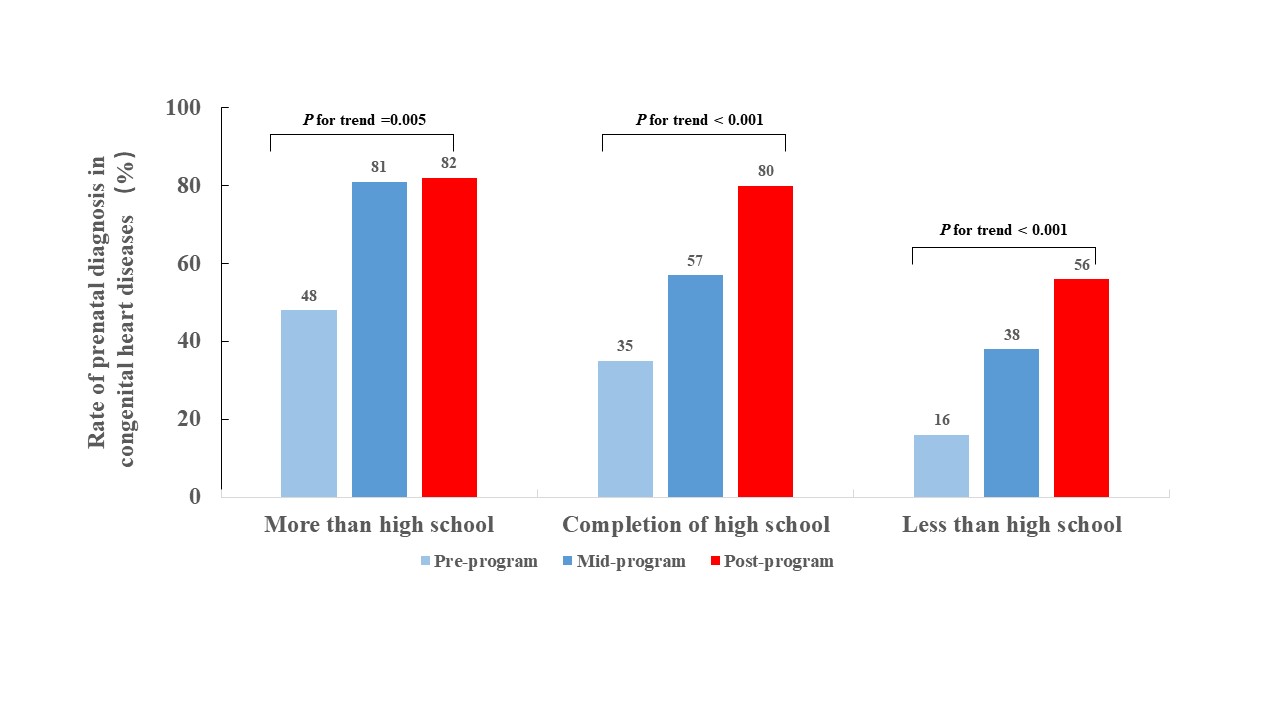

Supplement: Supplementary Figure 2 — Prenatal diagnosis rate for congenital heart disease by maternal education, 2004-2015. [file Image_2.jpg]

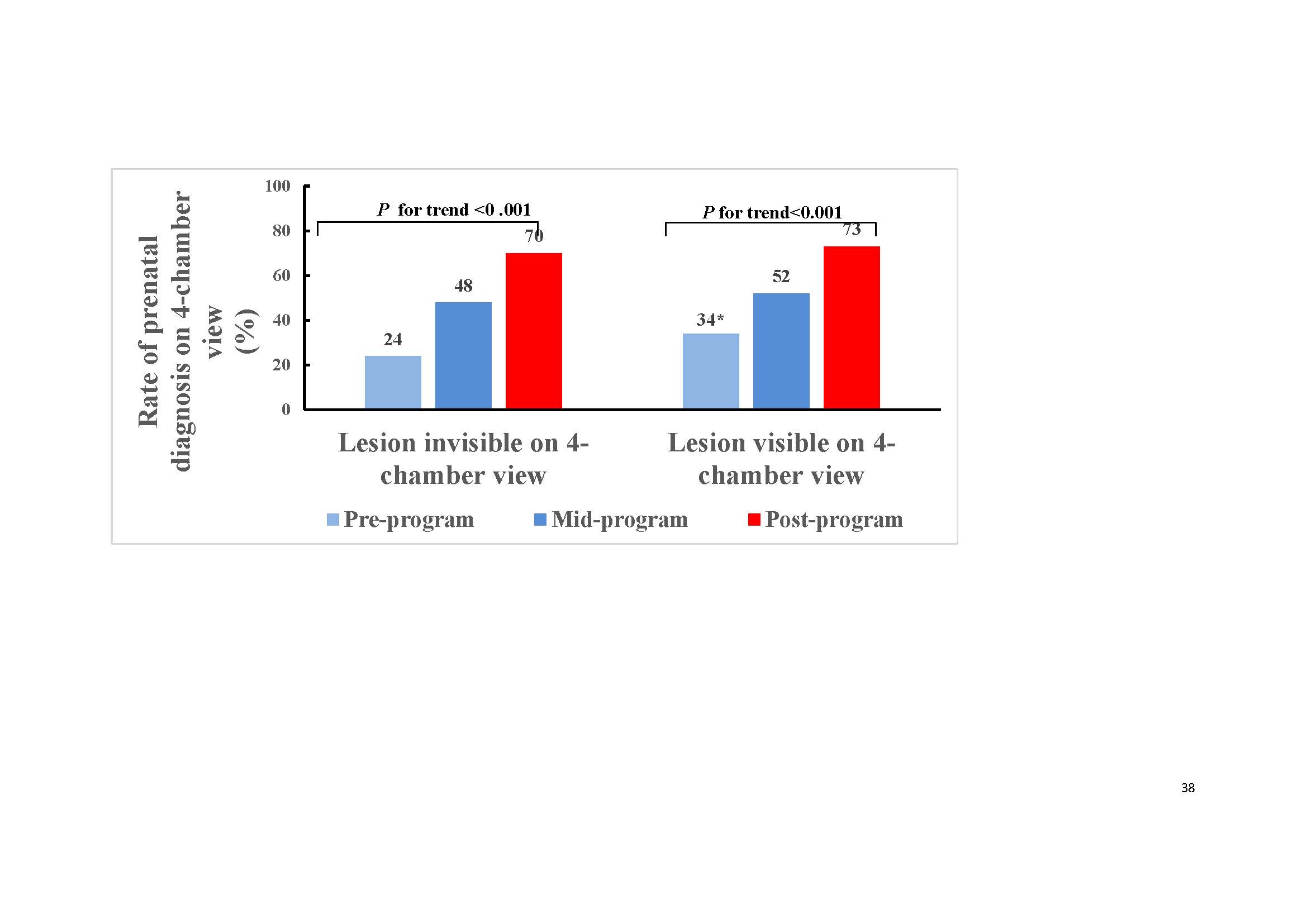

Supplement: Supplementary Figure 3 — Trend for prenatal diagnosis rates for congenital heart disease during pre-, mid- and post-program time intervals, by invisible or visible on the 4-chamber view of fetal ultrasound screening. *P < 0.05 compared to the pre-program PDR in lesion invisible on 4-chamber view. [file Image_3.JPEG]
