# Supplementary material for: Prenatal Detection of Congenital Heart Diseases Using Echocardiography: 12-Year Results of an Improving Program With 9782 Cases
Source: Front Public Health. 2022 May 13;10:886262. doi: 10.3389/fpubh.2022.886262 (PMC9136016; doi:10.3389/fpubh.2022.886262)
Supplement: Supplementary file 4 [file Data_Sheet_1.PDF]

Supplementary Materials

Supplementary Table 1. Odds of prenatal diagnosis for major CHD in different time-intervals.

Program Period	Adjusted OR (95% CI) in tertiary hospital	Adjusted OR (95% CI) in secondary hospital	Adjusted OR (95% CI) in primary hospital	P for interaction of counterpart × hospital level
Pre-program	(Reference)	(Reference)	--	<0.001
Mid-program	4.51 (2.54, 8.03)	9.53 (1.11, 81.48)	(Reference)	
Post-program	11.65 (6.52, 20.81)	20.95 (2.47, 178.06)	4.74 (0.41, 54.50)	
**Maternal age**				
>35 yrs	2.05 (1.04, 4.04)	1.86 (0.82, 4.19)	--	0.328
≤35 yrs	(Reference)	(Reference)	(Reference)	
**Domestic migrant population**				
Yes	0.46 (0.32, 0.66)	2.44 (1.60, 3.70)	--	0.003
No	(Reference)	(Reference)	(Reference)	
**Maternal education**				
more than high school	2.10 (1.35, 3.29)	1.13 (0.70, 1.84)	2.51 (0.34, 18.80)	0.080
completion of high school	1.76 (1.20, 2.57)	2.81 (1.25, 6.34)	--	
less than high school	(Reference)	(Reference)	(Reference)	
**Total previous live births**				
0	(Reference)	(Reference)	(Reference)	<0.001

1	0.10 (0.64, 0.17)	0.23 (0.14, 0.37)	0.28 (0.03, 3.13)	
2	0.03 (0.01, 0.09)	0.05 (0.01, 0.19)	--	
**Family history of CHD**				
Yes	0.46 (0.05, 4.21)	6.10 (0.42, 88.90)	--	0.726
No	(Reference)	(Reference)	(Reference)	
**Maternal diabetes**				
Yes	33.33 (3.84, ∞)	1.71 (0.51, 5.77)	--	0.627
No	(Reference)	(Reference)	(Reference)	
**Infant sex**				
female	9.57 (6.72, 13.63)	1.00 (0.66, 1.49)	0.51 (0.08, 3.09)	<0.001
male	(Reference)	(Reference)	(Reference)	
**Extra-cardiac/aneuploidy/genetic syndrome**				
Yes	1.91 (0.91, 4.00)	3.21 (1.69, 6.11)	8.10 (0.17, 388.55)	0.021
No	(Reference)	(Reference)	(Reference)	
**Fetus with suspected cardiac abnormality on basic obstetric ultrasound**				
Yes	∞	--	--	1.000
No	(Reference)	--	--	
**Maternal hypertension**				
Yes	7.12 (8.33, ∞)	0.76 (0.08, 7.42)	--	0.837
No	(Reference)	(Reference)	--	
